# Her Heart's Mistake

**Published:** 1889-08-10

**Authors:** E. D. Cumming

**Affiliations:** Author of "An Ordeal by Silence"


### Her Heart's Mistake.

By E. D. Cumming, Author of "An Ordeal by Silence."

*(Continued from page 285.)*

### Chapter XVII.—Her Eyes are Opened.

A soldier in karkee uniform and white helmet was standing before Miss Harrison in the verandah of the corner bungalow. He carried a large shallow box under his arm, and held a ponderous dogchain, to which a sad-looking monkey was attached. The man was pale and thin, having the appearance of one who has lately recovered from illness. He was one of Kate's hospital *protégés*, who had come for the joint purpose of paying her a visit of thanks and carrying out the wishes of a deceased comrade. She stood, resting one hand on the table at her side, listening to a somewhat lengthy address, with an expression of dismayed gratitude.

"And John Mullins sez to me, 'e sez, 'Wicks, if I dies you'll be my eggzegiter, an',' sez 'e, 'if you dies I'll be your eggzegiter,' 'e sez. An' I sez, 'Right you are, Mullins,' I sez, an' if it please you, ma'am, I've brought this 'ere monkey along to you."

Private Wicks paused for breath, and made a gesture with the chain, which caused the melancholy monkey to sprawl in an intoxicated fashion towards Kate. She involuntarily retreated a step, but the animal composed itself in a sitting attitude, scratched the small of its back, and eyed her with a solemn visage, apparently anxious to hear what she had to say.

"It's extremely kind of you, Wicks," she said, "and also of poor Mullins to think of me, but really I could not accept the monkey. I don't like to deprive you of your pet."

"'E was Mullinses' pet, ma'am; leastways 'e catched 'im at Jubbulpore, and my pals—H company, ma'am—they sez 'it's a pity,' they sez, 'a reel pity, but Wicks,' they sez, 'you're Mullinses' eggzegiter, you are, an' you're dooty bound to give 'im to the lady.'"

"Well then, Wicks, you must tell your friends that you carried out Mullins' wishes and brought me the monkey, but that I asked you to keep it and take care of it for me. It will be much happier with you than with me, for I should have to keep it in the stable where it would see no one and be very lonely."

Private Wicks weighed this aspect of the case thoughtfully for a few moments; it would have been a great trial to him and his comrades to part with the monkey, but he felt "dooty bound" to carry out the late owner's testamentary wishes. The lady did not wish to take it, and had referred in moving terms to the loneliness of the life to which it would be condemned in the stable. She was unquestionably right; no monkey of such tastes and accomplishments as this could exchange the cheerfulness and refinement of barrack life for the low society of black niggers and be happy.

"I'll take 'im back, then, ma'am?"

"Yes, if you will take care of him for me."

"I'll take hevery care of 'im ma'am," said the soldier with great earnestness; "and if you please, ma'am, I made so bold as to bring you these yer butterflies and scorpins, which I 'ope you'll take 'em."

The odour from the box he opened was not inviting, for some of the pin-impaled scorpions were neither fresh nor dried up; but the military entomologist who had spent many days in collecting them, presented the case with an air of would-be deprecating pride which told the value he set upon them. Kate accepted the collection, and in spite of the inconvenience suffered by her nose, spent a good five minutes admiring the butterflies and the taste with which they were arranged.

"Them's worth pounds in England, I'm told," said the collector blushing with pleasure at the young lady's praise, after the contents of the box had been minutely inspected and laid aside.

"And are you quite strong and well again now, Wicks?" she asked, after repeating her thanks, "I suppose your leg won't let you walk very far."

"I can git about now," said the convalescent, "but arter I come out o' 'orsepital I couldn't go no further nor the canteen for a bit."

He drew his cuff across his mouth with insinuating deliberation, and looked very hard at the monkey, which was holding its tail in both hands and examining the extremity with pensive interest.

"May I 'ave a drop of water for 'im, ma'am, if you please?" he enquired, wishing to convey his hint in the most delicate manner possible.

Kate smiled, and ordered water for the monkey and beer for the man. The former declined, and the latter accepted refreshment, and having assured his patroness that the monkey did not "live on the chain," in other words was not always confined by it, Private Wicks took the animal under his arm and departed, fraught with Bass and contentment.

Miss Harrison's labours at the hospital brought her many such visitors from time to time, and had she willed it, the house might have been filled with livestock of every description from parroquets to reclaimed pariahs. She made a beginning, and one which she often had reason to congratulate herself upon, by refusing Private John Mullins' well-intended legacy, and it became an understood thing in the lines that the Doctor's daughter preferred to receive her soldier friends empty-handed; albeit this conclusion was not fully arrived at until Corporal Fenwick's tame mongoose had been offered and declined. Kate's rejection of this zoological curiosity created great astonishment amongst the many men whose highest ambition was to own such an animal, and, according to their several dispositions, they decided that it would be safe or useless to lay their various pets at her feet.

Mr. Warren came to dinner that evening, and Kate, who quietly studied his demeanour during the meal, could see no trace of the manner which had led her to suppose she had offended him. He was as friendly and pleasant to her as usual, and when they adjourned to the verandah he made no attempt to leave her and join the Doctor, who, worn out by a hard day's work, had thrown himself into a long arm-chair, and seemed to have settled down for a nap. The night was cloudy and dark, and the verandah looked lighter than it generally did by comparison, when they took their seats below the punkah where the mosquitoes were least active.

"You may light your cheroot, Mr. Warren," said Kate. "I don't mind smoke; and if I did, I should have to accustom myself to it as one of a wife's duties. George is an inveterate smoker."

"Have you heard from him lately?" asked Warren, telling himself that he must learn to hear her speak of Bairdsley as her future husband, with indifference.

"I had a short note from him yesterday. He seems to spend his life going from one party to another."

"I believe that he is a man who is greatly liked in society," said Warren.

"He sings so beautifully, you know; but I don't think he goes in for popularity like some men."

"I often think that the fellows who cultivate universal popularity must be superficial and a little insincere. Of course, there are a few—dear old Phelps, for instance—whom everyone likes; but no one would think of accusing him of affectation or insincerity. But the regular society man's business is to be all things to all men."

"The society woman certainly makes it her business," said Kate.

"Not more so than men, only they take less pains to conceal, or have not the power to conceal, that it is only a manner."

The chaukidar was shuffling round the bungalow, his shoes grating noisily on the path as he walked, redoubling the vehemence of his guttural cry, to impress his employers with his wakefulness. He emitted a prolonged shout as he passed the spot where Kate and Warren were sitting, and as the Doctor had gone to sleep the former called to him to cease his professional exclamations for the present.

"Your father seems tired to-night, Miss Harrison," said Warren, glancing at the still figure which lay in the chair with one half-opened hand resting on the cement floor of the verandah.

"Yes, you will excuse him, I'm sure; some of the medical staff are away, and he has even more to do than usual."

She looked towards him for a moment, and resumed her restful attitude to continue her conversation with Warren.

Neither of them had seen the ugly, wicked-looking head of a snake that was gliding silently and slowly round the corner from the side verandah. Neither of them saw the long, lithe body drawing gradually nearer and nearer the Doctor's chair.

"Your father never spares himself, Miss Harrison."

"No; I wish he would for his own sake."

The cobra stopped and raised its hideous head a few inches, as if to look around, while its tail quivered restlessly.

"He retires at the end of the year, does he not?"

"Yes; he can stay longer if he likes, but I don't think he will. He is very anxious to go home before he loses all his old friends there."

The snake glided a few feet nearer the sleeper's chair and stopped again.

"I think it would be well for some of us if we come out to India with the fixed intention of adopting it as our home."

"I don't believe anyone could quite crush out the longing to return, or banish the idea that they are to do so one day. It would be against Nature."

The cobra was under the Doctor's chair now, and had once more lifted its head in surprise at the apparition of the white hand, which hung loosely on the floor. Its beady eyes were fixed upon the unwonted sight, and the head moved rapidly from side to side, as the black, forked tongue darted from between its horny lips like a spring. Death was below his chair, but the Doctor slept on; and the others did not see it.

"You will go home, too, I suppose, before very long. The 110th has nearly finished its term of foreign service, I understand."

"That will depend upon George. I hope he will not want to remain out here."

They talked in low tones, that they might not disturb the Doctor's rest, and neither cast a glance in his direction. He was sleeping very soundly. The cobra came nearer the hand, and glided over the half-closed fingers, steadily, stealthily, as though about to climb into the low-seated chair. It were better that the sleeper should never wake again than waken now: he holds his life in his hand in very truth, for the cobra's fangs will be buried in his wrist if he move a finger. Better that he should draw his last breath now while his thoughts are in far away England, than unseal the eyelids that must close for ever if they open now. May God help him who comes nearer death than this. Your faithful self-sacrifice stands you in good stead to-night, Doctor; such sleep as yours comes only to the weary.

Warren and Kate had been sitting in silence for a few minutes, and presently the former threw away the glowing end of his cheroot, and sat upright preparatory to bidding the young lady good night.

"Don't wake your father," he said, as he rose to his feet.

Kate glanced at the Doctor, and saw his danger for the first time. She sprang up and clutched Warren's arm like a vice, pointing to the hand on the floor, her face livid with terror; and for an instant the two stood paralysed.

Warren was the first to recover himself.

"Chloroform!" he said, in a hoarse whisper. "Is there any in the house?"

His presence of mind nerved the girl and she flew into her father's room, where there was a range of shelves holding various medicines and drugs. She could not think; she never asked herself whether chloroform would be amongst them, but ran her eyes over the rows of phials until they showed her what she sought. She snatched the bottle from the shelf, and was back at Warren's side twenty seconds after she had left him. He had bound his handkerchief round the end of a cane he had taken from the whip-rack on the wall, and almost before she had pressed the phial into his hand he had the stopper out and was drenching the handkerchief with the drug.

The cobra, coiled round the sleeper's wrist, was swaying its head backwards and forwards with the regular precision of a pendulum. Was it going to strike in sheer wantonness? Warren moved softly towards the Doctor's chair with the chloroformed handkerchief held well out before him, while Kate pressed her hands together, and looked on with burning eyes. She saw the cobra draw back its head to strike, and its hood expand; she heard its angry hiss, as he thrust the handkerchief against its head. She saw the scaly brown folds relax, and watched the deadly reptile slip from its victim's hand, overpowered by the anæsthetic, hardly realising what she saw. And then the overtaxed nerves gave way, and she fell fainting to the ground.

As the fatal bracelet fell from his arm, the Doctor sighed in his sleep, and raised his hand to his head.

"Another three seconds and it would have been all over with him," thought Warren as he snatched the handkerchief from the cane, and relieved his pent-up feelings by dealing the senseless snake a few well-directed blows.

"Halloa! What are you doing?" enquired Dr. Harrison, sitting upright, aroused by the energetic strokes.

"I'll explain in a minute," said Warren, dropping his stick. "We had better look after Miss Harrison now."

"What does this mean?" asked her father anxiously, rising from his chair and casting his eyes in the direction Warren indicated. "Merciful God! was that under my chair?" he exclaimed as he saw the cobra.

"It was round your wrist, sir," said the barrister. There! I can't tell you now. Let us look after Miss Harrison."

They raised her and laid her on a lounge, but before the Doctor returned with the restoratives he went to bring she had come to herself again, to find Warren bending over her.

"He is safe," he said, as she opened her eyes. For a moment they looked at one another in silence; then Warren turned away, and left the house without a word.

*(To be continued.)*

